# Does the data tell the true story? A modelling assessment of early COVID-19 pandemic suppression and mitigation strategies in Ghana

**DOI:** 10.1371/journal.pone.0258164

**Published:** 2021-10-29

**Authors:** Nana Kena Frempong, Theophilus Acheampong, Ofosuhene O. Apenteng, Emmanuel Nakua, John H. Amuasi

**Affiliations:** 1 Department of Statistics and Actuarial Science, College of Science, Kwame Nkrumah University of Science and Technology (KNUST), Kumasi, Ghana; 2 Department of Economics, University of Aberdeen, Aberdeen, Scotland, United Kingdom; 3 Research Group for Genomic Epidemiology, National Food Institute, Technical University of Denmark; 4 Department of Epidemiology and Biostatistics, School of Public Health, Kwame Nkrumah University of Science and Technology (KNUST), Kumasi, Ghana; 5 Department of Global Health, College of Science, Kwame Nkrumah University of Science and Technology (KNUST), Kumasi, Ghana; 6 Kumasi Center for Collaborative Research in Tropical Medicine (KCCR), Kumasi, Ghana; 7 Bernhard Nocht Institute of Tropical Medicine (BNITM), Hamburg, Germany; Institute for Advanced Sustainability Studies, GERMANY

## Abstract

This paper uses publicly available data and various statistical models to estimate the basic reproduction number (*R*_*0*_) and other disease parameters for Ghana’s early COVID-19 pandemic outbreak. We also test the effectiveness of government imposition of public health measures to reduce the risk of transmission and impact of the pandemic, especially in the early phase. R_0_ is estimated from the statistical model as 3.21 using a 0.147 estimated growth rate [95% C.I.: 0.137–0.157] and a 15-day time to recovery after COVID-19 infection. This estimate of the initial R_0_ is consistent with others reported in the literature from other parts of Africa, China and Europe. Our results also indicate that COVID-19 transmission reduced consistently in Ghana after the imposition of public health interventions—such as border restrictions, intra-city movement, quarantine and isolation—during the first phase of the pandemic from March to May 2020. However, the time-dependent reproduction number (R_t_) beyond mid-May 2020 does not represent the true situation, given that there was not a consistent testing regime in place. This is also confirmed by our Jack-knife bootstrap estimates which show that the positivity rate over-estimates the true incidence rate from mid-May 2020. Given concerns about virus mutations, delays in vaccination and a possible new wave of the pandemic, there is a need for systematic testing of a representative sample of the population to monitor the reproduction number. There is also an urgent need to increase the availability of testing for the general population to enable early detection, isolation and treatment of infected individuals to reduce progression to severe disease and mortality.

## 1 Introduction

The Coronavirus disease (COVID-19), a novel infectious disease caused by the Severe Acute Respiratory Syndrome coronavirus 2 (SARS-CoV-2), was declared a global pandemic by the World Health Organization (WHO) on March 11, 2020 [1, 2]. The first reported case was in December 2019 in Wuhan, the capital of China’s Hubei province, from where it quickly spread globally, reaching Sub-Saharan Africa (SSA), including Ghana [3, 4]. Ghana reported its first case of COVID-19 on March 12, 2020. Since then, the Government of Ghana had reported 91,009 total confirmed cases with 88,810 recoveries and 752 deaths as of April 07, 2021 [5, 6]. In response to the outbreak, the Ghanaian government, like other sovereign nation-states, imposed public health measures such as restrictions on movement, including lockdowns to contain the spread of the virus. The country closed land, air and sea borders in mid-March 2021 [7–10]. In addition, all educational institutions, including universities, high schools, and primary schools (both public and private), were closed [7–10]. The authorities also imposed bans on public gatherings. On April 20, 2020, the government lifted a three-week lockdown on the movement of persons within some parts of the Greater Accra, Tema, Kasoa and Greater Kumasi metropolis [7–10].

Preliminary evidence of the impact of these restrictions on the mobility patterns of populations for the period between February 17 and May 03, 2021, using anonymised and aggregated data from Ghana’s Mobile Network Operators (MNOs), indicated that person movements decreased up to 60% relative to the baseline value (Ghana Statistical Service, 2020). For example, in the Greater Accra Region, where about 70% of the total cases were located, person movements decreased by 20–30% during the period when initial restrictions were put in place compared to the baseline value [11]. This further decreased by 50–60% during the 3-week lockdown period, relative to the baseline level. A similar trend was observed for the Kumasi Metropolis, which had 15% of the total cases at that time [11].

There is a natural expectation that the lockdown and other control measures would have had some positive effect in restricting movement and physical interaction, thereby possibly slowing the spread of the virus. However, there is a need to fully explore, using data, what the direction of impact and magnitude of these interventions truly was, especially in the early stages of the COVID-19 pandemic in Ghana. The emphasis on the early period of the pandemic covering March to July 2020 is significant because, within this period, Ghana was among the few countries in Africa actively testing suspected cases and their contacts via a Tracing, Testing, and Treatment (‘3Ts’) programme and publishing the positivity rate [10, 12]. For example, as of April 25, 2020, Ghana had the highest COVID-19 test per capita in SSA with a rolling 7-day average of 0.18 daily new COVID-19 tests per 1,000 people compared to South Africa’s 0.13 and Senegal’s 0.03 [13]. Some of the response strategies, such as tracing and testing contacts, were subsequently scaled back, largely due to financing constraints and the continued spread. Nevertheless, data from this early period (March to July 2020) is relatively more accurate and reliable as reflecting the epidemiology of the disease in Ghana than that collected much later. In this early period, testing was centralized and compliance with timely reporting was high.

To formulate a valid and reliable estimate of transmissibility and spread of the COVID-19 outbreak in Ghana, we have utilised statistical modelling, which facilitates understanding of the mathematical determination of how the disease spreads. In epidemiological studies, several mathematical and statistical models have been proposed to understand the transmission dynamics of infectious diseases [14–18]. Modelling an infectious disease such as COVID-19 in an African country with reliable data is critical to understanding the transmission dynamics. This knowledge can be applied to planning and decision-making at various levels.

To assess the intensity of an outbreak, transmissibility can be quantified by the basic reproduction number (R_0_), that is, the average number of secondary infections generated by a single primary infective in a completely susceptible population [19–21]. The importance of estimating the basic reproduction number (R0) becomes more apparent when an emerging infectious disease strikes a population and there is a need for a tailored intervention. Monitoring the basic reproduction number provides insight into how the spread of COVID-19 is impacted by various events or interventions and is critical for decision-making in introducing or modifying (including lifting) social-distancing measures and their health and socio-economic implications [22–27]. R_0_ is, therefore, the fulcrum of any predictive modelling regarding Ghana’s COVID-19 epidemic curve, including any effort to estimate the impact of expected events which modify human movement and contact and most importantly, any mitigation strategies. The R_0_ is therefore one of governments’ key metrics for determining how well otherwise disruptive disease-spread mitigation strategies like lockdowns (of various forms) and other policies worked and might work in the future [28, 29]. We can also monitor the R_0_ to determine if these polices are worth the socio-economic costs they impose, especially in a largely informal economy such as Ghana’s [30]. R_0_ is a dynamic value that can vary geographically and temporally [31, 32]. It can be different from one day to another and from one place to another for the same disease, and its increase or decrease is determined by local conditions and human behaviour [33, 34].

Given the foregoing, this paper aims to estimate the basic reproduction number (R_0_) and other disease parameters for the early evolution of the COVID-19 pandemic in Ghana. We also use our estimations to explain the epidemiology of the disease in Ghana and explore the effectiveness of the government’s imposition of public health measures to suppress and mitigate the further spread of the pandemic in the country from March to July 2020. We develop a statistical model for the initial COVID-19 outbreak using various parametric growth models for the daily new cases in Ghana based on publicly available data. Additionally, we estimated the evolution of the reproduction number before a local maximum incidence of the COVID-19 pandemic in the country. Two methods are used to estimate the evolving R_0_, namely Sequential Bayesian (SB) and Time-Dependent (TD). The Jack-knife estimation technique is also applied to the data to examine any inconsistencies in testing rates within the period under review.

## 2 Materials and methods

### 2.1 Data sources

**Fig 1:**
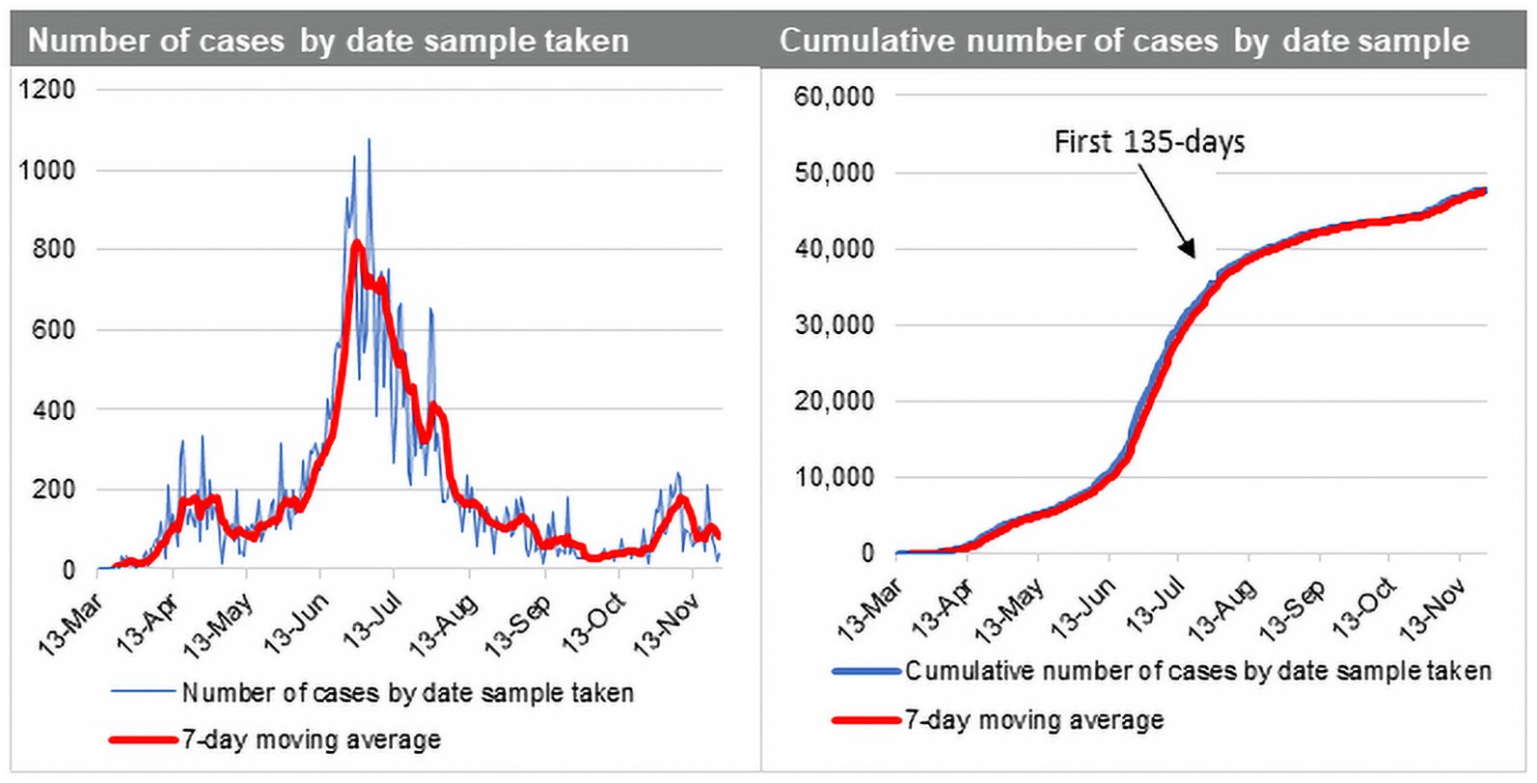
Ghana COVID-19 daily cases (Mar-Dec 2020).

Data for the COVID-19 pandemic in Ghana was obtained from the Ghana Health Service, an agency operating under the Ministry of Health, Government of Ghana. The first 135 days after first case was reported (March 12, 2020 to July 24, 2020) was used for the analysis as the first phase of the epidemic in Ghana. Fig 1 shows COVID-19 indicators for Ghana from March to December 2020.

### 2.2 Statistical model for the initial COVID-19 outbreak in Ghana

We focus on the initial stage of the epidemic using daily incidence data from the date of first reported case in the country. The weekly reported cases used was to attenuate any potential bias introduced by reporting delays. We let *Y(t)* be the number of COVID-19 cases at time *t*. We assume that

Y(t)~Poisson(λ(t)) (1)

where *λ*(*t*) is the mean number of cases per unit time.

We assume that in the initial stages of the epidemic, the daily incidence cases follow some growth models. The following are the models considered to estimate the growth rate of the initial stage of the epidemic.


$$\lambda \left(t\right)=\{\begin{array}{ll} g_{0}e^{\theta t} & \text{Exponential}\;\text{linear}\;\text{model}\\ g_{0}t^{\theta } & \text{Weibull}\;\text{model}\\ g_{0}\frac{\alpha e^{\theta t}}{1+\alpha e^{\theta t}} & \text{Logistic}\;\text{growth}\\ g_{0}\left(1-e^{-\alpha e^{\theta t}}\right) & \text{Gompertz}\;\text{growth} \end{array}$$ (2)

We note that at *t* > 0, the initial number of cases *g*_0_ is a constant and used as a parameter in the model. The parameter represents the growth rate of the epidemic. An estimate of the growth rate would help estimate the basic reproduction number for the proposed mathematical model of COVID-19 [35].

#### 2.2.1 Estimating initial R_0_ under a Generalised Linear Model (GLM) framework

The number of COVID-19 cases at time *t* modelled falls under the GLM framework [36] with Poisson distribution for the response and a log link function. The exponential linear and Weibull models in Eq (2) would be formulated under the GLM while the log-logistic and Gompertz growth models are formulated under non-linear models. Using the log link, we transformed the mean function of both exponential and Weibull models to a linear structure. We assume the number of cases *y*(*t*_*i*_) at time *t*_*i*_ is independently Poisson distributed with mean $\lambda_{i}=g_{0}e^{\theta t_{i}}$.

The log-likelihood function is given as:

$$l\left(g_{0},\theta \right)=\overset{n}{\underset{i=1}{\sum }}ln\frac{e^{-\lambda_{i}}\lambda_{i}^{y_{i}}}{y_{i}!}=\overset{n}{\underset{i=1}{\sum }}-\lambda_{i}+y_{i}\;ln\;\lambda_{i}-ln\;y_{i}!$$ (3)

Maximizing the log-likelihood ignoring terms independent on the parameters and substituting *λ*_*i*_ in (3).


$$\left({\hat{g}}_{0},\hat{\theta }\right)=\underset{\left({\overset{⌢}{g}}_{0},\hat{\theta }\right)}{argmax}\overset{n}{\underset{i=1}{\sum }}-\lambda_{i}+y_{i}\;ln\;\lambda_{i}=\underset{\left({\overset{⌢}{g}}_{0},\hat{\theta }\right)}{argmax}\overset{n}{\underset{i=1}{\sum }}-g_{0}e^{\theta t_{i}}+y_{i}\;ln\;g_{0}+\theta y_{i}t_{i}$$ (4)

Similarly, for $\lambda_{i}=g_{0}t_{i}^{\theta }$ the log-likelihood is given as

$$\left({\hat{g}}_{0},\hat{\theta }\right)=\underset{\left({\overset{⌢}{g}}_{0},\hat{\theta }\right)}{argmax}\overset{n}{\underset{i=1}{\sum }}-\lambda_{i}+y_{i}\;ln\;\lambda_{i}=\underset{\left({\overset{⌢}{g}}_{0},\hat{\theta }\right)}{argmax}\overset{n}{\underset{i=1}{\sum }}-g_{0}t_{i}^{\theta }+y_{i}\;ln\;g_{0}+\theta y_{i}\;ln\;t_{i}$$ (5)

For $\lambda_{i}=g_{0}\left(1-e^{-\alpha e^{\theta t_{i}}}\right)$, the log-likelihood is given as

$$\left({\hat{g}}_{0},\hat{\alpha },\hat{\theta }\right)=\underset{\left({\overset{⌢}{g}}_{0},\hat{\alpha },\hat{\theta }\right)}{argmax}\overset{n}{\underset{i=1}{\sum }}-\lambda_{i}+y_{i}\;ln\;\lambda_{i}=\underset{\left({\overset{⌢}{g}}_{0},\hat{\alpha },\hat{\theta }\right)}{argmax}\overset{n}{\underset{i=1}{\sum }}-g_{0}\left(1-e^{-\alpha e^{\theta t_{i}}}\right)+y_{i}\;ln\;g_{0}+y_{i}\;ln\;\left(1-e^{-\alpha e^{\theta t_{i}}}\right)$$ (6)

For $\lambda_{i}=g_{0}\frac{\alpha e^{\theta t_{i}}}{1+\alpha e^{\theta t_{i}}}$, the log-likelihood is given as

$$\left({\hat{g}}_{0},\hat{\alpha },\hat{\theta }\right)=\underset{\left({\overset{⌢}{g}}_{0},\hat{\alpha },\hat{\theta }\right)}{argmax}\overset{n}{\underset{i=1}{\sum }}-\lambda_{i}+y_{i}\;ln\;\lambda_{i}=\underset{\left({\overset{⌢}{g}}_{0},\hat{\alpha },\hat{\theta }\right)}{argmax}\overset{n}{\underset{i=1}{\sum }}-g_{0}\frac{\alpha e^{\theta t_{i}}}{1+\alpha e^{\theta t_{i}}}+y_{i}\;ln\;g_{0}+y_{i}\;ln\;\lambda_{i}$$ (7)

This maximisation problem is solved numerically using the Newton-Raphson’s algorithm.

The basic reproduction number *R*_0_ is closely related to the initial growth rate *θ* under the assumption of exponentially distributed latent and infectious periods using the formula [37]:

$$R_{0}=\frac{\beta }{\gamma },\;\theta =\beta -\gamma ;\;where\;\theta =\left(R_{0}-1\right)\gamma$$ (8)

where *β*, *γ* are the transmission and recovery rates respectively.

#### 2.2.2. Estimating R_0_ over time before a local maximum incidence of COVID-19

Two methods were considered for the estimation of *R*_*0*,_ namely sequential Bayesian (SB) and Time- Dependent (TD). These two methods rely on the generation time, which is the time lag between infection in a primary case and transmission to a secondary case. The generation time distribution is obtained from the time lag between all infective/infector pairs, as it cannot be observed directly from the data. So, we often substitute with the serial interval distribution that measures time between symptom onset. The implementation was done in the R software using the *R*_*0*_ package implemented by Obadia et al. [38]. These methods rely on the assumption that the number of secondary cases caused by an index case is Poisson distributed. Given observation of (*N*_0_, *N*_1_, …, *N*_*T*_) incident cases over consecutive time units, and a generation time distribution w, *R*_*0*_ is estimated by these two methods:

*2*.*2*.*2*.*1 Sequential Bayesian method (SB)*. This method relies on an approximation to the SIR model [39–42] whereby incidence at time *t* + 1, *N*(*t* + 1) is approximately Poisson distributed with mean

$$\mu_{t}=N\left(t\right)e^{\left(\gamma \left(R-1\right)\right)}$$ (9)

Bayesian framework starts with a non-informative prior on the distribution of the reproduction number *R*_*0*_. The distribution is updated as new data is observed, using

$$P(R|N_{0},N_{1}\ldots ,N_{t+1})=\frac{P(N_{t+1}|R,N_{0},\ldots N)P(R|N_{0},\ldots ,N_{t})}{P\left(N_{0},\ldots ,N_{t+1}\right)}$$ (10)

*2*.*2*.*2*.*2 Estimation of time-dependent reproduction numbers (TD)*. The time-dependant method proposed by Wallinga & Teunis [43], computes reproduction numbers by averaging over all transmission networks compatible with observations. The probability *p*_*ij*_ that case *i* with onset at time *t*_*i*_ was infected by case *j* with onset at time *t*_*j*_ is calculated as

$$p_{ij}=\frac{N_{i}w\left(t_{i}-t_{j}\right)}{\sum_{i\neq k}N_{i}w\left(t_{i}-t_{k}\right)}$$ (11)

The effective number for case *j* is therefore *R*_*j*_ = ∑_*j*_ *p*_*ij*_ and averaged as $R_{t}=\frac{1}{N_{t}}\sum_{\left\{t_{j}=t\right\}}R_{j}$ over all cases with same date of onset.

#### 2.2.3 Estimating the impact of easing restrictions on infection rates

Fokianos and Fried [44] proposed intervention time series and models interventions [45] affecting the location by including a deterministic covariate and a decay rate. Following Fokianos and Fried [44, 45] we let *Y*_*t*_ be the number of COVID-19 cases at time *t*. We assume that *Y*_*t*_ ~ *NegBin* (*λ*_*t*_, *ϕ*), where *λ*(*t*) is the mean number of cases per unit time and *ϕ* the dispersion parameter. In general. the linear predictor of a model with *s* types of interventions according to parameters *δ*_1_, …, *δ*_*s*_ occurring at time points *τ*_1_, …, *τ*_*s*_ where *w*_*m*_, *m* = 1, …, *s* are intervention sizes is given as follows:

$$g\left(\lambda_{t}\right)=\beta_{0}+\overset{p}{\underset{k=1}{\sum }}\beta_{k}\tilde{g}\left(Y_{t}{}_{-i_{k}}\right)+\overset{q}{\underset{l=1}{\sum }}\alpha_{l}\tilde{g}\left(\lambda_{t}{}_{-i_{l}}\right)+\eta^{T}X_{t}+\overset{s}{\underset{m=1}{\sum }}w_{m}\delta_{m}^{t-\tau_{m}}Ι(t\geq \tau_{m})$$ (12)

The maximum likelihood estimation (MLE) is used to obtain the parameter estimates.

By identifying interventions and estimating their effect size, we allow for the identification of structural changes which can be assessed using valid statistical inferences. That is, did the said intervention have an effect on a time series during the respective period(s) in question. This is a test under the null hypothesis (H_0_) of no intervention, against the alternative hypothesis (H_1_) of some intervention at a known time τ. If the policy interventions worked, then we should see a statistically significant sign on the coefficient estimates (betas). These betas are then exponentiated to get the multiplicative rate: anything above 1 indicates increase in infection rates, and those below 1 indicate a decrease in infection rates. We assume the following: no seasonality variations (daily time series data), a level shift effect (between days 5–10, rate of change of infection could be negative or positive but need to test statistical significance) for each intervention, and in some cases, the external effects of the interventions are accounted for.

## 3 Results and discussion

### 3.1 Estimating the true incidence rate by correcting for inconsistencies in testing rates

**Fig 2:**
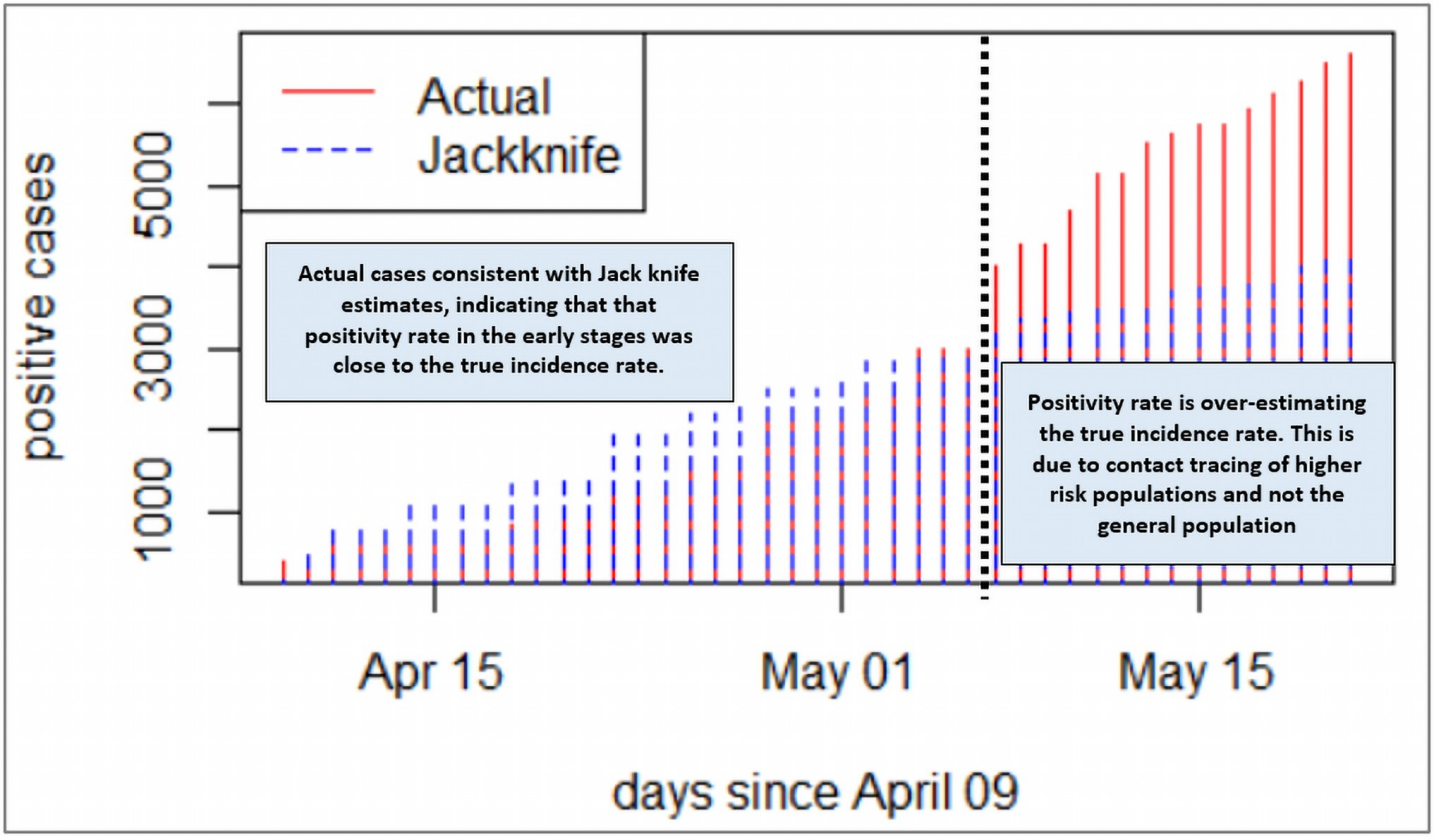
Jackknife estimates versus actual number of positive cases.

The Jack-knife method of estimating the incidence rate of COVID-19 infection has been applied to the data to correct for any inconsistencies in testing rates within the period under review [46, 47]. Ideally, to calculate the population (true) incidence rate, one needs to have consistent sampling rate (preferably per day) from a representative sample of the population, in which case the positivity rate would be expected to mirror the incidence rate [48–51]. From Fig 2, the Jack-knife estimation is consistent with the actual COVID-19 case numbers observed in Ghana until May 7, 2020. The number of positives recorded is considerably higher than the Jack-knife estimates. This departure is consistent with the increased number of tests conducted on contact traced individual and concentrated within hotspots around that period, and neither general nor representative population level sampling. Thus, with increased testing targeted at hotspots and contacts of persons who tested positive, one would expect a higher number of positive cases. This indicates a critical need to have consistency in testing rates and sampling representative of the population to generate a true incidence rate and reproduction number. The forementioned estimates would form the basis of monitoring the impact of control interventions, informing critical policy decisions. In this regard, an exclusive focus on the actual number of positives after May 7 as the basis for measuring the state of affairs with regard to COVID-19 spread could be erroneous and lead to a false alarm.

### 3.2 Initial reproduction number (*R*_*0*_)

Several growth models for the initial outbreak of COVID-19 in Ghana were considered and presented in Table 1.

**Table 1:** Estimates of different parameter models.

Growth Model	Estimates (standard errors)	Akaike Information Criterion (AIC)
Exponential	0.095 (0.002)**	-16914.29
Weibull	2.15 (0.062)**	-16946.64
Logistic	0.17 (0.006)**	-16991.13
Gompertz	0.147 (0.0051)**	-17011.42

** significant at 5%

The estimated models are shown below:

$$\hat{\lambda }\left(t\right)=\{\begin{array}{ll} 4.9e^{0.095t} & \text{Exponential}\;\text{linear}\;\text{model}\\ 0.06t^{2.15} & \text{Weibull}\;\text{model}\\ 190\frac{0.008e^{0.17t}}{1+0.008e^{0.17t}} & \text{Logistic}\;\text{growth}\;\text{model}\\ 169.07\left(1-e^{-0.01e^{0.147t}}\right) & \;\text{Gompertz}\;\text{growth}\;\text{model} \end{array}$$

From Table 1, the underlying assumption behind the models is that in the initial stage of the epidemic, the number of infected individuals emerges at an exponential rate. We observe that the estimated growth rate for each model is statistically significant at 5% with the Gompertz model having the minimum AIC although all models were significantly close. The estimated growth rate from this model is 0.147 with 95% C.I. [0.137–0.157]. From Eq (8), assuming a 15-day time to recovery [52] after COVID-19 infection, the initial reproduction number is estimated as 3.205. That is: *R*_*O*_ = 1 + (0.147 * 15) = 3.21. The United States’ Centers for Disease Control and Prevention (CDC) [52] reported 95% of laboratory-confirmed SARS-CoV-2 specimens tested did not yield replication-competent virus after 15 days following symptom onset.

Table 2 shows our estimates of the initial reproduction number for Ghana compared with other countries. Ghana’s initial reproduction number is very close to that reported for South Africa and Morocco and relatively close to Kenya, Algeria but further afield to figures from some Chinese provinces and South Korea [55–64]. This result is also note-worthy for one key reason: during the early days of the pandemic, Ghana was testing relatively larger numbers than several African countries at a good rate by adopting a pooled sampling testing approach, which allowed a lot more COVID-19 tests to be conducted [53, 54]. Ghana, for example, is reported as having conducted over 370,000 tests between March and mid-July 2020, putting the country among the highest in Africa with regard to number of tests when standardised by the population size [53]. Generally speaking, countries testing at a good rate were getting R_0_ greater in the high 2s and low 3s.

**Table 2:** Estimates of R_0_ and comparison to other country studies.

Country	Initial Reproduction Number (R0) Estimate	Source
Ghana	3.21	Authors’ estimate
South Africa	3.20	[55]
Egypt	2.30	[56]
	2.29	[55]
Algeria	2.66	[55]
Morocco	2.99	[57]
Kenya	2.82	[55]
Nigeria	2.29	[55]
China	4.03 (Hubei Province)	[58]
	3.80 (Wuhan)	[59]
	2.60 (Wuhan)	[60]
	2.68 (Wuhan)	[61]
	2.20 (Wuhan)	[62]
	2.61 (China)	[63]
South Korea	3.47–3.54 (Daegu/NGP)	[58]
United Kingdom	2.6	[64]

### 3.3 Time dependent and Sequential Bayesian reproduction number

**Fig 3:**
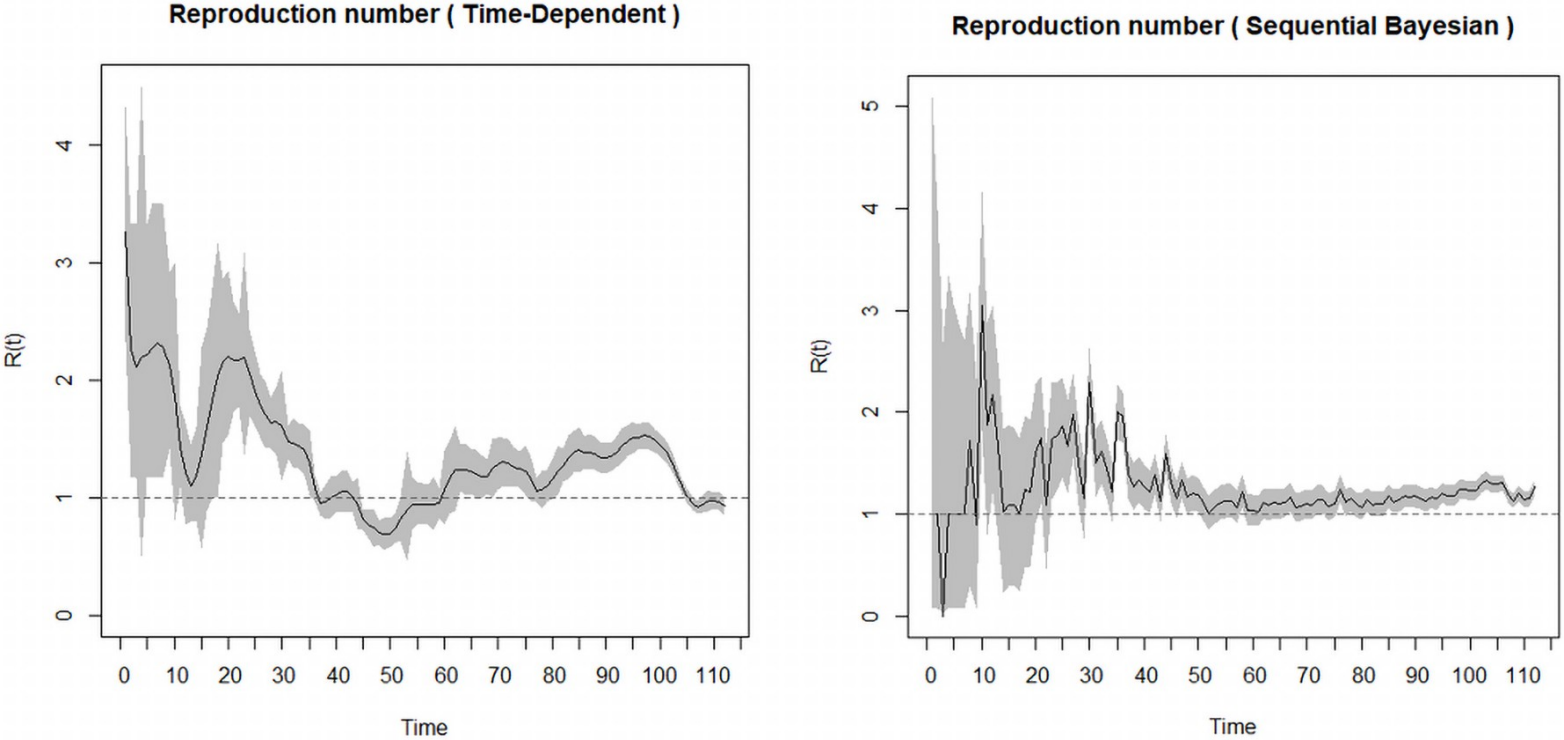
(a) Time dependent and (b) Sequential Bayesian reproduction number.

It will be necessary for the generation time distribution to be discretised using the same time unit. We therefore assumed a Gamma distribution for the generation time distribution with mean 3.57 and standard deviation 2.55 [65, 66] when there are control measures in place. We also assumed that the infectious period is the same for asymptomatic and symptomatic cases. These methods require a period over which there is an exponential growth. The period chosen is the date from first case(s) (March 12, 2020; day 1) to the date of maximum incidence (April 25, 2020; day 44) (Fig 3). From the two outputs in Fig 3, the Sequential Bayesian does not make biological reasoning because the assumption of equal probabilities of *R*_*0*_ does not hold [42]. Hence, we opt for the time-dependent method.

**Fig 4:**
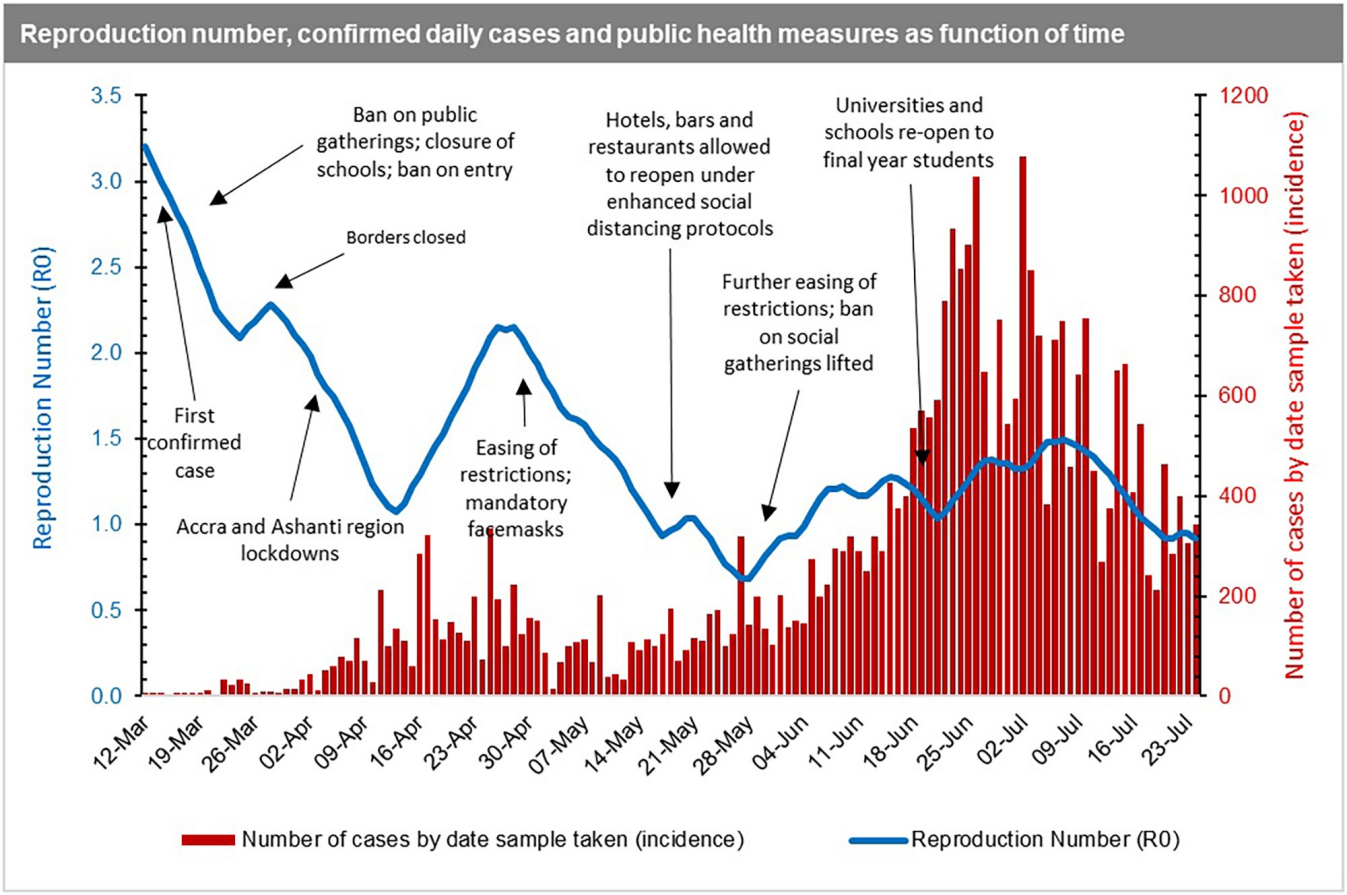
Time dependent R_0_ superimposed on daily COVID-19 infections in Ghana.

From Fig 4, the first estimates of *R*_*0*_ in Ghana reflect the high rate of spread at the onset (March 12, 2020) of the pandemic. This was right before the mandatory quarantine of all persons entering the country, including events such as the ban on public gatherings, closure of schools, churches, mosques, and restriction of entry into Ghana for anyone who had been in a country with more than 200 COVID-19 cases. The spread of the disease measured by the *R*_*0*_ then declines after those key events from the first reported date (March 12, 2020) to the date of border closure (10 days interval). Between the date of border closure and right before the announcement of the partial lockdown of two regions (Greater Accra and Greater Kumasi), which reported more than 90% of the cases, the spread of the disease increased moderately. After the initial lockdown, the spread of the disease reduced initially but begun to pick up momentum again from mid-April onwards with the easing of restrictions. This was despite the introduction of the wearing of mandatory facemasks, which were not fully complied with by citizens due to the largely informal nature of settlements [30] in the country making enforcement even more challenging.

**Fig 5:**
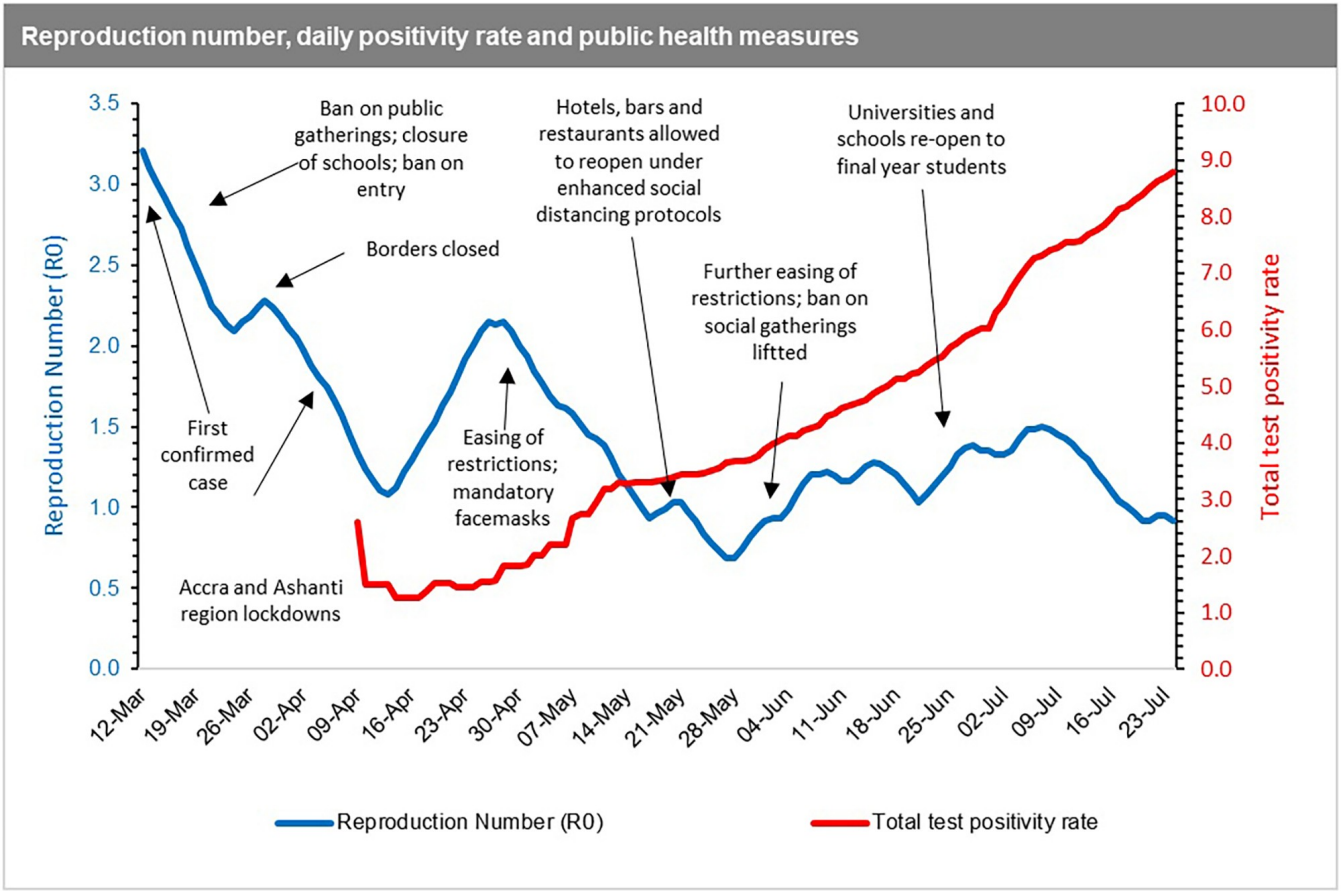
Time dependent R_0_ superimposed on daily positivity rate in Ghana.

In essence, the reproduction number reduces from March until mid-April during the lockdown, and then upsurged again when the country relaxed the initial restrictions. However, we note that the *R*_*0*_ decreases further even after restrictions are eased (including when bars and hospitality venues were allowed to operate). With this observation, it can be inferred that the reproduction number and incidence rate paint an accurate picture of the COVID-19 spread in Ghana at the early stages of the pandemic from March to early May 2020. In these early stages, Ghana’s actual cases of COVID-19 are consistent with the Jack-knife estimates (see Fig 2), indicating that the positivity rate in the early stages was close to the true incidence rate. Beyond the early stages of the pandemic, it becomes difficult to make any deductive inferences based on the data available which might reflect the true situation. Our argument is further supported by the fact that contrasting to the *R*_*0*_, the positivity rate, which ideally should be a proxy for the incidence rate, had been on the ascendency since mid-April (Fig 5).

In essence, the reproduction number estimated will be unreliable if a country does not have a consistent and population representative testing regime. In Ghana’s case, the reproduction number exhibits significant volatility as can be seen in the time-dependent charts (Figs 4 and 5), indicating that it is not representative of the true progression of the epidemic, as there was not a consistent testing regime in place. The reproduction number is a function of the frequency of potential exposures, and not having a consistent testing regime in place means that any such estimate is subject to misrepresentation and will be ineffective for use in evidence-based policy making [22, 33, 67–70]. In fact, a very high positivity rate might actually indicate that a country is not doing enough COVID-19 testing within the general population to find and isolate as many cases as possible to curb transmission.

### 3.4 Impact of easing restrictions on COVID-19 infection rates

**Fig 6:**
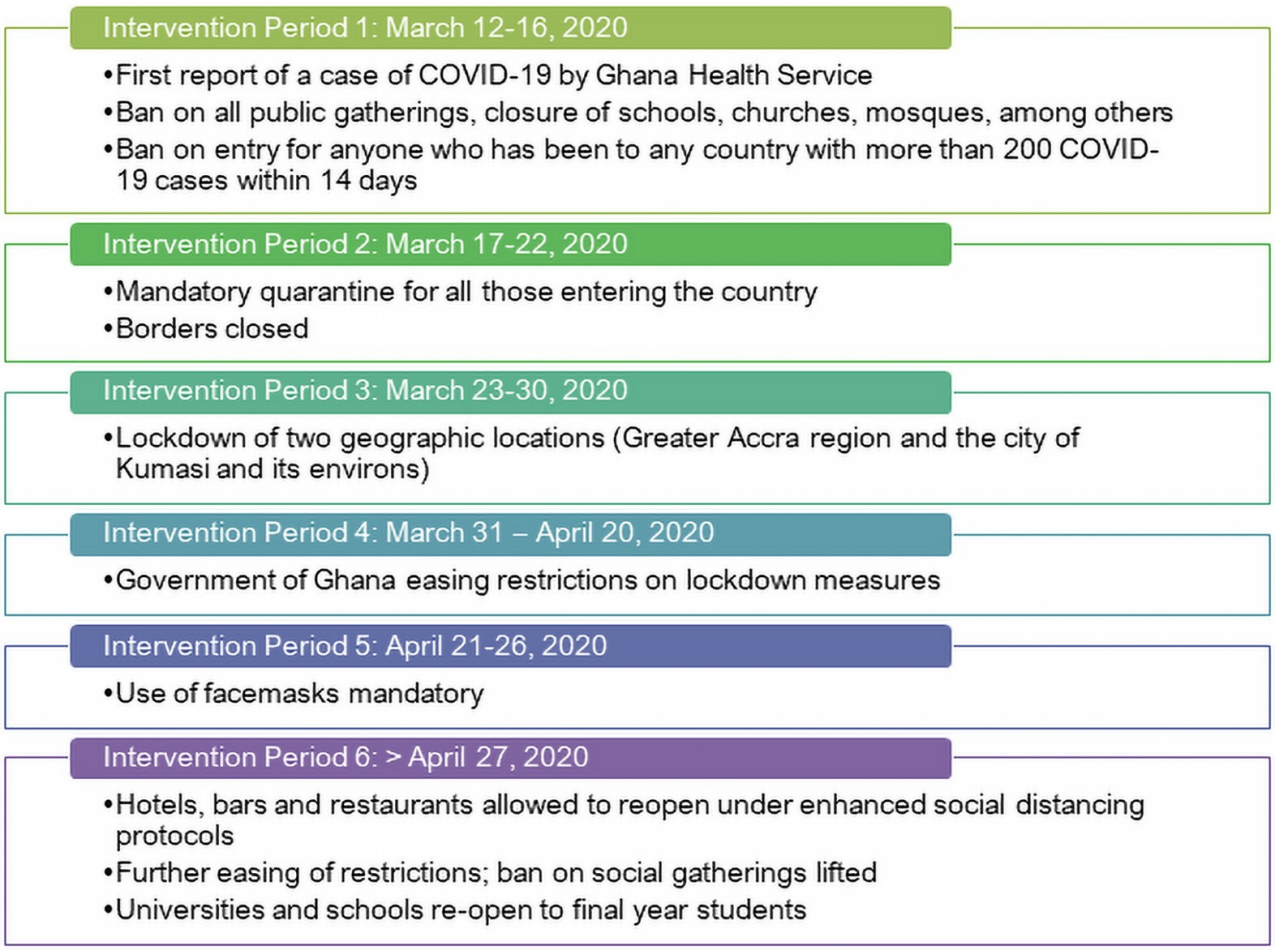
Summary of intervention periods and public health measures imposed.

Next, we look at the intervention periods (Fig 6) and their mitigation effect on the rate of infections. As discussed in **Section 2.2.3**, we fitted a Negative-Binomial model with the log link function by considering serial dependence, which includes a regression on the two successive previous observations, and includes the six intervention effects detected by the model [71]. Using this approach, we can assess the effect of interventions such as easing restrictions on COVID-19 infection rates and separate these from general trends and serial dependencies in time [72].

As can be seen in Table 3, there is a mixed a story of the impact of the mitigation effects of easing restrictions on COVID-19 infection rates. The initial three weeks following the confirmation of the first case on March 12 was characterised by a 25% increase in infection rate although this is not statistically significant (Intervention Period 1). During this time, the Ghana government swiftly moved to impose a ban on all public gatherings, closure of schools, churches, mosques, among others. It also banned entry for anyone who has been to any country with more than 200 COVID-19 cases within 14 days. Intervention Period 2 was accompanied by the policy announcement of the mandatory quarantine for all those entering the country and the closure of air and land borders. The period was characterised by a 6% decrease in infection rate, but this was again not statistically significant impact.

**Table 3:** Testing for intervention effects. The null hypothesis (H_o_) is no intervention effect.

Date	Intervention Type	Time (days)	Coeff. Estimates (95% Confidence Interval)	Multiplicative effect (rate of infection)	Comment (Below 1 = decrease; Above 1 = increase)
March 12–16	Intervention Period 1	5<intervention< = 10	**0.223**	Exp (0.223) = 1.250	25% increase in the rate of infection. However, there is no significant impact
			**[-0.676; 1.130]**		
March 17–22	Intervention Period 2	10<intervention< = 18	**-0.066**	Exp (-0.066) = 0.936	6% decrease in the rate of infection. No significant impact
			**[-0.829; 0.683]**		
March 23–30	Intervention Period 3	18<intervention< = 38	**0.445**	Exp (0.445) = 1.560	56% increase in the rate of infection. However, this is not statistically significant.
			**[-0.083; 1.035]**		
March 31 –April 20	Intervention Period 4	38<intervention< = 44	-0.382***	Exp (-0.382) = 0.682	Statistically significant 32% decline in rate of infection.
			**[-1.003; -0.145]**		
April 21–26	Intervention Period 5	44<intervention< = 79	**0.129**	Exp (0.129) = 1.138	14% increase in rate of infection. However, this not statistically significant impact
			**[-0.325; 0.716]**		
>27 April	Intervention Period 6	Intervention>79	**0.043**	Exp (0.043) = 1.044	4% increase in rate of infection. However, this not statistically significant impact
			**[-0.163; 0.283]**		

Source: Authors’ estimates | *** highly significant (<0.001)

Following this, Intervention Period 3 saw the government move to further impose the lockdown of Ghana’s main population centres: the two geographic locations—Greater Accra Region (5.06 million) and the city of Kumasi and its environs (3.4 million residents)—which collectively makes up about 27% of the country’s 31 million population as of 2020. At the time of Intervention Period 3, we also see a 56% decrease in infection rate. However, this is not statistically a significant impact. The Ghana government begun easing restrictions on these earlier lockdown measures imposed by Intervention Period 4. However, we observe a statistically significant 32% decline in rate of infection relative to previous intervention period. We explain this trend as possibly due to the residual positive effect of the preceding intervention period now kicking in, or due to a lag in the actual resumption of mass gatherings, as all had been cancelled and were either yet to be rescheduled or simply did not come off any longer. These are also confirmed in several studies, notably Meiksin [73], Li et al. [74] and Shengjie et al. [75]. For example, Meiksin [73] notes in the context of the United Kingdom that the weekly death rate of registered COVID-19 deaths peaked just over three weeks after the partial lockdown and continued to decline. Li et al. [74] also found reduced COVID-19 transmission following the introduction of non-pharmaceutical interventions such as school closure, workplace closure and public events ban of between 3% to 24% on day 28 (four weeks) following the introduction, compared with the last day before introduction. Likewise, Shengjie et al. [75] found that non-pharmaceutical interventions deployed in China were effective in containing the COVID-19 outbreak. However, the efficacy of the different interventions was dependent on early case detection and effective contact reduction.

Increases in infection rate also occasioned intervention Periods 5 and 6 as many citizens began to fully get back to their normal activities and despite the use of facemasks now made mandatory. For example, hotels, bars and restaurants being allowed to reopen under enhanced social distancing protocols as part of further easing of restrictions. Additionally, the ban on social gatherings was lifted and universities and schools were re-open to final year students.

## 4 Conclusions

The data available suggests that the COVID-19 lockdown and other restrictions in Ghana did reduce movement and interaction, which would positively slow down the spread of the virus in the early phase of the pandemic. This conclusion is further supported by anonymised and aggregated data from Mobile Network Operators on the mobility patterns of populations published by the Ghana Statistical Service for the period between February 17 and May 03, 2020 showing that person movements decreased by up to 60% relative to the baseline value during the period when restrictions were in place.

The initial reproduction number (*R*_*0*_) is estimated from the statistical model as 3.21 using a 0.147 estimated growth rate and a 15-day time to recovery after COVID-19 infection. We observe that the estimated growth rate for each model is significant at 5%, with the Gompertz model having the minimum AIC, although all models were significantly close. The estimate of the initial *R*_*0*_ is consistent with what is reported in the literature from other parts of Africa, China and Europe. The *R*_*0*_ reflects the high rate of spread at the outset of the pandemic in the country before the mandatory quarantine of all persons entering the country and the initial ban on public gatherings, among other public health interventions. The *R*_*0*_ estimates and incidence rates could be seen as a fair reflection of the COVID-19 epidemiology at the early stages of the pandemic in Ghana (from March to early May 2020). From our analysis, Ghana’s actual cases reported are consistent with the bootstrap Jack-knife estimates, indicating that positivity rate in the early stages closely mirrored the incidence rate.

Going forward, there is a need for more robust, consistent, systematic and representative sampling for testing, first to enable early detection, isolation and treatment of infected individuals and to reduce progression to severe disease and risk of mortality. Second, our recommended testing regime is critical for generating data for monitoring the impact of interventions to inform policy decisions, especially in this period of looming austerity. We therefore recommend the widespread deployment of point-of-care diagnostics and later rapid diagnostic tests (RDT) when they become more reliable, coupled with timely reporting and dissemination of results. This will serve as a first-line strategy towards mass surveillance of the population both to monitor the reproduction number and estimate the true incidence rate of COVID-19. The fore-mentioned measures are not only logistically less burdensome but would also prove timelier and more cost effective than running full-sample or pooled PCR tests.

Monitoring the impact of control measures on the evolution of the pandemic in Ghana will require being able to determine any changes in transmission rates, hospitalization rates and death rates over time. Each of these measures provide unique insights for disease control and prevention policy making and implementation but can only be determined by consistent, systematic and population representative testing mechanisms. Authorities would therefore need to rapidly set up a nationally representative monitoring systems involving sentinel sites, including schools, markets, places of worship, mass transit stations and other places of gathering in each of the 16 regions of Ghana. From these sentinel sites, a regular number of tests via an algorithm will need to be conducted (preferably on a daily basis), with results made available within 24-48hrs. Results from this system could serve as the index for measuring the infectivity rate and subsequently, an accurate-enough time-dependent *R*_*0*_ on a daily and weekly basis in different parts of the country.

While the results presented in this paper provide important insights into the early evolution of the pandemic in Ghana, we note that Ghana’s daily incidence data at the early stages which has been used in the analysis, was sparse, lacked granularity and did not show a consistent trend. This means our assumptions of the disease parameter estimates must be applied cautiously, while improvements in sampling and testing strategies are improved as we have recommended.

## References

[1] ReferencesCucinotta D, Vanelli M. WHO declares COVID-19 a pandemic. Acta Bio Medica: Atenei Parmensis. 2020;91(1):157. doi: 10.23750/abm.v91i1.9397 32191675 PMC7569573

[2] World Health Organization. Pandemic (Covid19)—Update. (2020a). https://www.who.int/emergencies/diseases/novel-coronavirus-2019 (Accessed: February 10 2021).

[3] Leung K, Wu JT, Liu D, Leung GM. First-wave COVID-19 transmissibility and severity in China outside Hubei after control measures, and second-wave scenario planning: a modelling impact assessment. The Lancet. 2020;395(10233):1382–93. 10.1016/S0140-6736(20)30746-7 PMC7195331 32277878

[4] Cowling BJ, Ali ST, Ng TW, Tsang TK, Li JC, Fong MW, et al. Impact assessment of non-pharmaceutical interventions against coronavirus disease 2019 and influenza in Hong Kong: an observational study. The Lancet Public Health. 2020 May 1;5(5):e279–88. doi: 10.1016/S2468-2667(20)30090-6 32311320 PMC7164922

[5] Africa CDC. COVID-19 Daily Updates; 2021. https://africacdc.org/covid-19 (Accessed: April 9 2021).

[6] Ghana Health Service. COVID-19 Updates; 2020. https://www.ghanahealthservice.org/covid19/archive.php (Accessed: February 15 2021).

[7] Ghana: Authorities ban entry to travelers from countries with more than 200 COVID-19 confirmed cases as of March 17 /update 1; 2020. https://www.garda.com/crisis24/news-alerts/323076/ghana-authorities-ban-entry-to-travelers-from-countries-with-more-than-200-covid-19-confirmed-cases-as-of-march-17-update-1 (Accessed: July 25 2021).

[8] March 21 2020—Akufo-Addo shuts down Ghana’s borders; 2020. https://www.ingsa.org/covid-19-policy-tracker/africa/ghana/21-march-2020-akufo-addo-shuts-down-ghanas-borders (Accessed: July 25 2021).

[9] Quakyi N.K.; Asante N.A.A.; Nartey Y.A.; Bediako Y.; Sam-Agudu N.A. Ghana’s COVID-19 response: The Black Star can do even better. BMJ Glob. Health 2021, 6, e005569. doi: 10.1136/bmjgh-2021-005569 33712435 PMC7958583

[10] Zhang, J., Nonvignon, J., Mao, W. How well is Ghana—with one of the best testing capacities in Africa—responding to COVID-19?; 2020, Brookings. https://www.brookings.edu/blog/future-development/2020/07/28/how-well-is-ghana-with-one-of-the-best-testing-capacities-in-africa-responding-to-covid-19 (Accessed: February 15 2021).

[11] Ghana Statistical Service. Mobility analysis to support the Government of Ghana in responding to the COVID-19 outbreak; 2020. https://statsghana.gov.gh/COVID-19%20press%20release%20report%20-%20analysis%20overview%20-%20final.pdf (Accessed: February 15 2021).

[12] Sibiri H, Prah D, Zankawah SM. Containing the impact of COVID-19: Review of Ghana’s response approach. Health policy and technology. 2020 November 4. doi: 10.1016/j.hlpt.2020.10.015 33169101 PMC7641588

[13] OurWorldInData. Coronavirus Pandemic Data Explorer; 2020. https://ourworldindata.org/coronavirus-data-explorer?zoomToSelection=true&time=2020-03-01.latest&country=GHA~ZAF~KEN~NGA~SEN&region=World&testsMetric=true&interval=smoothed&perCapita=true&smoothing=7&pickerMetric=total_cases&pickerSort=desc (Accessed: February 15 2021).

[14] Krämer A, Kretzschmar M, Krickeberg K, editors. Modern infectious disease epidemiology: Concepts, methods, mathematical models, and public health. Springer Science & Business Media; 2010

[15] Diekmann O, Heesterbeek JA. Mathematical epidemiology of infectious diseases: model building, analysis and interpretation. John Wiley & Sons; 2000.

[16] Brauer F, Castillo-Chavez C, Castillo-Chavez C. Mathematical models in population biology and epidemiology. New York: Springer; 2012.

[17] Gilbert M, Pullano G, Pinotti F, Valdano E, Poletto C, Boëlle PY, et al. Preparedness and vulnerability of African countries against importations of COVID-19: a modelling study. The Lancet. 2020 March 14;395(10227):871–7. doi: 10.1016/S0140-6736(20)30411-6 32087820 PMC7159277

[18] Diop BZ, Ngom M, Biyong CP, Biyong JN. The relatively young and rural population may limit the spread and severity of COVID-19 in Africa: a modelling study. BMJ global health. 2020 May 1;5(5):e002699. doi: 10.1136/bmjgh-2020-002699 32451367 PMC7252974

[19] Diekmann O, Heesterbeek JA, Metz JA. On the definition and the computation of the basic reproduction ratio R 0 in models for infectious diseases in heterogeneous populations. Journal of mathematical biology. 1990 Jun;28(4):365–82. doi: 10.1007/BF00178324 2117040

[20] Nishiura H. Mathematical and statistical estimation approaches in epidemiology. Chowell G, Hyman JM, Bettencourt LM, Castillo-Chavez C, editors. Dordrecht: Springer Netherlands; 2009 Dec 1.

[21] Chowell G, Nishiura H, Bettencourt LM. Comparative estimation of the reproduction number for pandemic influenza from daily case notification data. Journal of the Royal Society Interface. 2007 Feb 22;4(12):155–66. doi: 10.1098/rsif.2006.0161 17254982 PMC2358966

[22] Ma J, Dushoff J, Bolker BM, Earn DJ. Estimating initial epidemic growth rates. Bulletin of mathematical biology. 2014 January 1;76(1):245–60. doi: 10.1007/s11538-013-9918-2 24272389

[23] Eikenberry SE, Mancuso M, Iboi E, Phan T, Eikenberry K, Kuang Y, et al. To mask or not to mask: Modeling the potential for face mask use by the general public to curtail the COVID-19 pandemic. Infectious Disease Modelling. 2020 January 1;5:293–308. doi: 10.1016/j.idm.2020.04.001 32355904 PMC7186508

[24] Danon L, Brooks-Pollock E, Bailey M, Keeling MJ. A spatial model of CoVID-19 transmission in England and Wales: early spread and peak timing. MedRxiv. 2020. 10.1098/rstb.2020.0272 PMC8165591 34053261

[25] Hellewell J, Abbott S, Gimma A, Bosse NI, Jarvis CI, Russell TW, et al. Feasibility of controlling COVID-19 outbreaks by isolation of cases and contacts. The Lancet Global Health. 2020 Apr 1;8(4):e488–96. doi: 10.1016/S2214-109X(20)30074-7 32119825 PMC7097845

[26] Wilson N, Barnard LT, Kvalsig A, Verrall A, Baker MG, Schwehm M. Modelling the potential health impact of the COVID-19 pandemic on a hypothetical European country. medRxiv. 2020a.

[27] Wilson N, Barnard LT, Kvalsvig A, Baker M. Potential health impacts from the COVID-19 pandemic for New Zealand if eradication fails: report to the NZ Ministry of Health. Ministry of Health; 2020b.

[28] Rypdal M, Rypdal V, Jakobsen PK, Ytterstad E, Løvsletten O, Klingenberg C, et al. Modelling suggests limited change in the reproduction number from reopening Norwegian kindergartens and schools during the COVID-19 pandemic. PloS one. 2021 Feb 25;16(2):e0238268. doi: 10.1371/journal.pone.0238268 33630842 PMC7906341

[29] Santamaría L, Hortal J. COVID-19 effective reproduction number dropped during Spain’s nationwide dropdown, then spiked at lower-incidence regions. Science of the Total Environment. 2021 January 10;751:142257. doi: 10.1016/j.scitotenv.2020.142257 33181975 PMC7480327

[30] Danquah M, Schotte S. COVID-19 and the socioeconomic impact in Africa: The case of Ghana. InWIDER Background Note 2020/5 2020 May. Helsinki UNU-WIDER.

[31] Ridenhour B., Kowalik J.M. and Shay D.K., 2018. Unraveling r 0: Considerations for public health applications. American journal of public health, 108(S6), pp.S445–S454. 10.2105/AJPH.2013.301704 PMC3935673 24328646

[32] Ng TC, Wen TH. Spatially adjusted time-varying reproductive numbers: understanding the geographical expansion of urban dengue outbreaks. Scientific reports. 2019 Dec 16;9(1):1–2. 31844099 10.1038/s41598-019-55574-0 PMC6914775

[33] Delamater PL, Street EJ, Leslie TF, Yang YT, Jacobsen KH. Complexity of the basic reproduction number (R0). Emerging infectious diseases. 2019 Jan;25(1):1. doi: 10.3201/eid2501.171901 30560777 PMC6302597

[34] Dharmaratne S, Sudaraka S, Abeyagunawardena I, Manchanayake K, Kothalawala M, Gunathunga W. Estimation of the basic reproduction number (R0) for the novel coronavirus disease in Sri Lanka. Virology Journal. 2020 Dec;17(1):1–7. 33028382 10.1186/s12985-020-01411-0 PMC7538841

[35] Ma J. Estimating epidemic exponential growth rate and basic reproduction number. Infectious Disease Modelling. 2020 January 1;5:129–41. doi: 10.1016/j.idm.2019.12.009 31956741 PMC6962332

[36] McCullagh P., Nelder J.A. Generalized linear models. Chapman & Hall, London; 1989

[37] Feng Z. Final and peak epidemic sizes for SEIR models with quarantine and isolation. Mathematical Biosciences & Engineering. 2007;4(4):675. doi: 10.3934/mbe.2007.4.675 17924718

[38] Obadia T, Haneef R, Boëlle PY. The R0 package: a toolbox to estimate reproduction numbers for epidemic outbreaks. BMC medical informatics and decision making. 2012 Dec;12(1):1–9. doi: 10.1186/1472-6947-12-147 23249562 PMC3582628

[39] Hong HG, Li Y. Estimation of time-varying reproduction numbers underlying epidemiological processes: A new statistical tool for the COVID-19 pandemic. PloS one. 2020 Jul 21;15(7):e0236464. doi: 10.1371/journal.pone.0236464 32692753 PMC7373269

[40] Kypraios T, O’Neill PD. Bayesian nonparametrics for stochastic epidemic models. Statistical Science. 2018 Feb;33(1):44–56.

[41] Lai CC, Hsu CY, Jen HH, Yen AM, Chan CC, Chen HH. The Bayesian Susceptible-Exposed-Infected-Recovered model for the outbreak of COVID-19 on the Diamond Princess Cruise Ship. Stochastic Environmental Research and Risk Assessment. 2021 Jan 26:1–5. doi: 10.1007/s00477-020-01968-w 33519302 PMC7837082

[42] Clancy D, O’Neill PD. Bayesian estimation of the basic reproduction number in stochastic epidemic models. Bayesian Analysis. 2008 Dec;3(4):737–57.

[43] Wallinga J, Teunis P. Different epidemic curves for severe acute respiratory syndrome reveal similar impacts of control measures. American Journal of epidemiology. 2004 Sep 15;160(6):509–16. doi: 10.1093/aje/kwh255 15353409 PMC7110200

[44] Fokianos K, Fried R. Interventions in INGARCH processes. Journal of Time Series Analysis. 2010 May;31(3):210–25. doi: 10.1111/j.1467-9892.2010.00657.x

[45] Fokianos K, Fried R. Interventions in log-linear Poisson autoregression. Statistical Modelling. 2012 Aug;12(4):299–322. doi: 10.1177/1471082X1201200401

[46] Overgaard M, Parner ET, Pedersen J. Asymptotic theory of generalized estimating equations based on jack-knife pseudo-observations. The Annals of Statistics. 2017 Oct;45(5):1988–2015.

[47] Rao JN, Shao J. Jackknife variance estimation with survey data under hot deck imputation. Biometrika. 1992 Dec 1;79(4):811–22.

[48] Subramanian R, He Q, Pascual M. Quantifying asymptomatic infection and transmission of COVID-19 in New York City using observed cases, serology, and testing capacity. Proceedings of the National Academy of Sciences. 2021 March 2;118(9). 10.1073/pnas.2019716118 PMC7936345 33571106

[49] Hilborne LH, Wagner Z, Cabreros I, Brook RH. Linking statistics with testing policy to manage COVID-19 in the community. American journal of clinical pathology. 2020 July 7;154(2):142–8. doi: 10.1093/ajcp/aqaa099 32520340 PMC7314266

[50] Mercer TR, Salit M. Testing at scale during the COVID-19 pandemic. Nature Reviews Genetics. 2021 Jul;22(7):415–26. doi: 10.1038/s41576-021-00360-w 33948037 PMC8094986

[51] Furuse Y, Ko YK, Ninomiya K, Suzuki M, Oshitani H. Relationship of Test Positivity Rates with COVID-19 Epidemic Dynamics. International journal of environmental research and public health. 2021 Jan;18(9):4655. doi: 10.3390/ijerph18094655 33925665 PMC8125747

[52] CDC. Interim Guidance on Duration of Isolation and Precautions for Adults with COVID-19. 2020. https://www.cdc.gov/coronavirus/2019-ncov/hcp/duration-isolation.html (Accessed: April 10 2021).

[53] World Health Organization. Pooling samples boosts Ghana’s COVID-19 testing. 2020b. https://www.who.int/news-room/feature-stories/detail/pooling-samples-boosts-ghana-s-covid-19-testing (Accessed: April 10 2021).

[54] Nyazika TK, Kaela R, Mugoni M, Musomekwa K, Kyei-Baafour E, Chiwanda S, et al. Implementation of antibody rapid diagnostic testing versus real-time reverse transcription-PCR sample pooling in the screening of COVID-19: a case of different testing strategies in Africa. MSphere. 2020 Jul 29;5(4):e00524–20. doi: 10.1128/mSphere.00524-20 32727861 PMC7392544

[55] Zhao Z, Li X, Liu F, Zhu G, Ma C, Wang L. Prediction of the COVID-19 spread in African countries and implications for prevention and control: A case study in South Africa, Egypt, Algeria, Nigeria, Senegal and Kenya. Science of the Total Environment. 2020 August 10;729:138959. 10.1016/j.scitotenv.2020.138959 PMC7182531 32375067

[56] El Desouky ED. Prediction of the Epidemic Peak of Covid19 in Egypt, 2020. medRxiv. 2020

[57] Hammoumi A, Qesmi R. Impact assessment of containment measure against COVID-19 spread in Morocco. Chaos, Solitons & Fractals. 2020 Nov 1;140:110231. doi: 10.1016/j.chaos.2020.110231 32863612 PMC7442897

[58] Choi S, Ki M. Estimating the reproductive number and the outbreak size of COVID-19 in Korea. Epidemiology and health. 2020;42. 32164053 10.4178/epih.e2020011 PMC7285447

[59] Read JM, Bridgen JR, Cummings DA, Ho A, Jewell CP. Novel coronavirus 2019-nCoV: early estimation of epidemiological parameters and epidemic predictions. MedRxiv. 2020. 10.1098/rstb.2020.0265 PMC8165596 34053269

[60] Anastassopoulou C, Russo L, Tsakris A, Siettos C. Data-based analysis, modelling and forecasting of the COVID-19 outbreak. PloS one. 2020 Mar 31;15(3):e0230405. doi: 10.1371/journal.pone.0230405 32231374 PMC7108749

[61] Wu JT, Leung K, Leung GM. Nowcasting and forecasting the potential domestic and international spread of the 2019-nCoV outbreak originating in Wuhan, China: a modelling study. The Lancet. 2020 February 29;395(10225):689–97. doi: 10.1016/S0140-6736(20)30260-9 32014114 PMC7159271

[62] Li Q, Guan X, Wu P, Wang X, Zhou L, Tong Y, et al. Early transmission dynamics in Wuhan, China, of novel coronavirus–infected pneumonia. New England journal of medicine. 2020 January 29. 10.1056/NEJMoa2001316 PMC7121484 31995857

[63] Wang K, Zhao S, Li H, Song Y, Wang L, Wang MH, et al. Real-time estimation of the reproduction number of the novel coronavirus disease (COVID-19) in China in 2020 based on incidence data. Annals of translational medicine. 2020 Jun;8(11). doi: 10.21037/atm-20-1944 32617309 PMC7327374

[64] Jarvis CI, Van Zandvoort K, Gimma A, Prem K, Klepac P, Rubin GJ, et al. Quantifying the impact of physical distance measures on the transmission of COVID-19 in the UK. BMC medicine. 2020 Dec;18(1):1–0. 32375776 10.1186/s12916-020-01597-8 PMC7202922

[65] Davies NG, Klepac P, Liu Y, Prem K, Jit M, Eggo RM. Age-dependent effects in the transmission and control of COVID-19 epidemics. Nature medicine. 2020 Aug;26(8):1205–11. doi: 10.1038/s41591-020-0962-9 32546824

[66] Davies NG, Kucharski AJ, Eggo RM, Gimma A, Edmunds WJ, Jombart T, et al. Effects of non-pharmaceutical interventions on COVID-19 cases, deaths, and demand for hospital services in the UK: a modelling study. The Lancet Public Health. 2020 Jul 1;5(7):e375–85. doi: 10.1016/S2468-2667(20)30133-X 32502389 PMC7266572

[67] Valentin JB, Møller H, Johnsen SP. The basic reproduction number can be accurately estimated within 14 days after societal lockdown: The early stage of the COVID-19 epidemic in Denmark. Plos one. 2021 Feb 16;16(2):e0247021. doi: 10.1371/journal.pone.0247021 33592029 PMC7886116

[68] Inglesby TV. Public health measures and the reproduction number of SARS-CoV-2. Jama. 2020 June 2;323(21):2186–7. doi: 10.1001/jama.2020.7878 32356869

[69] Ridenhour B, Kowalik JM, Shay DK. Unraveling R0: Considerations for public health applications. American journal of public health. 2018 Dec;108(S6):S445–54. 10.2105/AJPH.2013.301704 PMC3935673 24328646

[70] Nishiura H, Chowell G, Safan M, Castillo-Chavez C. Pros and cons of estimating the reproduction number from early epidemic growth rate of influenza A (H1N1) 2009. Theoretical Biology and Medical Modelling. 2010 Dec;7(1):1–3. doi: 10.1186/1742-4682-7-1 20056004 PMC2821365

[71] Fokianos K. Statistical analysis of count time series models: A GLM perspective. Handbook of discrete-valued time series, Handbooks of Modern Statistical Methods. 2015 May:3–28.

[72] Gilmour S, Degenhardt L, Hall W, Day C. Using intervention time series analyses to assess the effects of imperfectly identifiable natural events: a general method and example. BMC medical research methodology. 2006 Dec;6(1):1–9. doi: 10.1186/1471-2288-6-16 16579864 PMC1481564

[73] Meiksin A. Dynamics of COVID-19 transmission including indirect transmission mechanisms: a mathematical analysis. Epidemiology & Infection. 2020;148. doi: 10.1017/S0950268820002563 33092672 PMC7642914

[74] Li Y, Campbell H, Kulkarni D, Harpur A, Nundy M, Wang X, et al. for COVID UN. The temporal association of introducing and lifting non-pharmaceutical interventions with the time-varying reproduction number (R) of SARS-CoV-2: a modelling study across 131 countries. The Lancet Infectious Diseases. 2021 Feb 1;21(2):193–202. doi: 10.1016/S1473-3099(20)30785-4 33729915 PMC7581351

[75] Shengjie L, Ruktanonchai NW, Zhou L. Effect of non-pharmaceutical interventions for containing the COVID-19 outbreak in China. medRxiv preprint.2020.

